# Editorial: From West to East: Recent Advances in Psychometrics and Psychological Instruments in Asia

**DOI:** 10.3389/fpsyg.2022.875536

**Published:** 2022-03-24

**Authors:** Yiyun Shou, Hui-Fang Chen, Kazuhisa Takemura, Joseph Wu, Cheng-Ta Yang, Meng-Cheng Wang

**Affiliations:** ^1^Research School of Psychology, Australian National University, Canberra, ACT, Australia; ^2^Department of Social and Behavioural Sciences, City University of Hong Kong, Hong Kong, Hong Kong SAR, China; ^3^Department of Psychology, Waseda University, Tokyo, Japan; ^4^Graduate Institute of Mind, Brain and Consciousness, Taipei Medical University, Taipei, Taiwan; ^5^Department of Psychology, National Cheng Kung University, Tainan, Taiwan; ^6^Department of Psychology, Guangzhou University, Guangzhou, China

**Keywords:** Asian, psychological instrument, assessment, measurement, cross-cultural

Over the past several decades, researchers have become increasingly aware of cultural influences on psychological processes. Yet, the majority of what we know about psychology is still based on published research that, for the most part, has been conducted in Western Euro-American populations-more specifically, Western, educated, industrialized, rich, and democratic (WEIRD) populations. The issue of the generalizability and applicability of psychological theories outside of WEIRD populations is one that has been increasingly raised in the literature (e.g., Laajaj et al., 2019; Muthukrishna et al., 2020; Roberts et al., 2020). The lack of research in non-Western cultures has been partly attributed to methodological issues especially the lack of cross-culturally valid measurement tools.

Most psychological instruments are developed in Western cultural contexts. There are various challenges in the application of this sort of psychological research and methods that have been conducted and developed in the West to non-Western cultures, and, in particular, to Asian cultural contexts. These challenges include, but are not limited to, issues of language, incongruent modes of expressions, and different cultural/societal norms and cognitive styles.

The current special issue summarizes and promotes recent advances in psychometrics and cross-cultural measurements of psychology in Asian communities. The special issue includes 32 research articles across various measurement topics, including the validation of psychological instruments for Asian communities that were originally developed in Western cultures, developing indigenous assessments for Asian communities, and applying advanced quantitative tools to address psychometric issues that are essential for cross-cultural research.

## Validating Psychological Instruments in Asian Communities

A number of the papers in this collection are devoted to examining the validity and applicability of psychological assessments for Asian communities that were originally developed in Western cultures. These instruments cover essential psychological constructs and processes in various fields, including clinical (e.g., depression, Park et al.), organizational (emotional labor, Yang, Chen et al.; work-family balance, Eguchi et al.), social (Social capital, Hasan et al.), and educational (academic interest, Luo, Dang et al.) psychology.

Our collection also goes beyond adult communities. Western and Asian cultures differ in important ways for young people, including family structure and relationships, as well as educational environments. Having appropriate tools for the youth is essential for understanding the developmental nuances of psychological processes and disorders. Yang, Zhang et al. and Ren et al. validated the Youth Psychopathic Traits Inventory (YPI) in Chinese detained and community children. Both studies replicated a bifactor model structure of YPI and recommended that YPI can be useful in the youth samples. In the educational setting, Luo, Dang et al. demonstrated that the Academic Interest Scale for Adolescents had good validity and reliability among Chinese adolescents and Wang, Christensen et al. applied the Reduced Instructional Materials Motivation Survey (RIMMS) to identify secondary school students' motivation profiles in an adaptive learning setting.

While most of the measures demonstrated promising psychometric properties such as reliability and convergent validity when being applied to Asian communities, some authors also noted differences in factor structures and the applicability of individual items of some scales in Asian communities as compared to their Western counterparts. For example, Park et al. examined the reliability and factor structure of the Beck Depression Inventory-II (BDI-II) among Korean adults. Despite having excellent internal consistency and convergent validity, Park et al. noted that the original somatic-affective factor of BDI might have two separate dimensions for Korean adults: somatic and performance difficulty. Similarly, Jiang et al. administered the Depression Anxiety Stress Scale-21 to Chinese medical staff and found that a single factor structure was the most parsimonious representation of negative affects captured by the DASS-21. The authors concluded that the original three-factor model might provide limited discriminant validity for Chinese medical staff. Wang, Ren et al. reported that the factor structure of the Body Perception Questionnaire–Short Form (BPQ-SF) was similar to results from American samples, but also noted some individual items may need further consideration.

## Adapting and Developing Measures for Asian Contexts

Adapting and developing culturally appropriate measures is important when the original measures have limited applicability in Asian culture contexts. Our collection also contains studies that propose modified or new instruments that are appropriate for Asian communities.

Huang et al. were concerned with culture and the role it played in measuring family quality of life (FQol) in Chinese samples. The authors interviewed family members of children with developmental disabilities and developed a questionnaire of FQoL for this group of participants. The original scale consisted of 10 dimensions and was evaluated and validated using both exploratory and confirmatory factor analyses. A sample of 40 families and 845 families participated in the interview and validation study, respectively. The results convincingly demonstrated the importance of Chinese culture and parents' perception of education or rehabilitation for children with developmental disabilities.

Hasan et al. accounted for various factors, including social context and language, and developed a modified social capital assessment for a northern state in India. The newly adapted scale captures four unique constructs of social capital, including engagement with the community, social support, trust, and social cohesion. Yang, Chen et al. addressed the emotional labor phenomena and indicated that a culturally specific measurement tool was needed for the Chinese cultural context considering the significant impact of culture on emotional regulation and display rules. The authors applied a range of procedures including field observations, in-depth interviews, and qualitative survey analysis to develop the initial version of the self-report scale. The formal scale was then validated in multi-wave samples and demonstrated its appropriateness for the Chinese cultural context.

Wu and Siu addressed concerns about problematic mobile phone use in adolescents and developed an instrument that was suitable for adolescents in Hong Kong. In their study, a new group of “at-risk” adolescents was identified by applying a criterion-referenced approach. The authors recommended that adolescents who are vulnerable to this technology-related problem should receive more attention from professionals. This study also echoes a recent call for adopting a developmental perspective in understanding this problem and conducting research in this area.

## Advanced Quantitative Methods

This special issue also features the application of various advanced quantitative methods to address psychometric issues in measurements. Traditionally, researchers often use confirmatory factor analysis to examine if the original factor structure of a scale can be applied to different cultures. A number of papers in this special issue also applied item response theory to further investigate item properties and to better understand item-level information. For instance, starting from a conventional approach, Dai et al. conducted confirmatory factor analysis (CFA) to examine if Chinese versions of the Interpersonal Trust Scale (ITS), Philosophies of Human Nature Scale (RPHNS), and Company Trust Scale (CTS) show the same factor structures proposed in the original scales. Findings suggested that factor structures varied in Chinese samples and thus the authors validated them again via item response theory and further evaluated reliability and item parameters in the Chinese scales.

Tian and Dai developed a computerized adaptive test (CAT) application to measure stress (termed CAT-S). They combined 226 items from 7 scales to develop an initial item bank, of which 93 items were retained in the final scale. The Perceived Stress Scale (PSS) was then used to examine the convergent validity of the CAT-S, which provides an efficient method to measure stress in Chinese college students. Luo, Cai et al. also developed a CAT measure to assess narcissistic personality using an item bank of 85 items among Chinese undergraduate students.

## Conclusions and Future Directions

In summary, the current special issue provides a showcase for recent advances in psychological measurements in Asian cultural contexts. Validation and adaptation are essential to achieve accurate assessment of target psychological constructs in Asian communities. The development of appropriate tools and investigating cultural impact from a psychometric perspective is essential for understanding psychological processes beyond the Euro-American context. It will also be important in the future to address these qualitative differences in culture from an axiomatic and representative measurement theory perspective.

## Author Contributions

All authors listed have made a substantial, direct, and intellectual contribution to the work and approved it for publication.

## Conflict of Interest

The authors declare that the research was conducted in the absence of any commercial or financial relationships that could be construed as a potential conflict of interest.

## Publisher's Note

All claims expressed in this article are solely those of the authors and do not necessarily represent those of their affiliated organizations, or those of the publisher, the editors and the reviewers. Any product that may be evaluated in this article, or claim that may be made by its manufacturer, is not guaranteed or endorsed by the publisher.
